# *Hyssopus cuspidatus Boriss* Volatile Extract (SXC): A Dual-Action Antioxidant and Antifungal Agent Targeting *Candida albicans* Pathogenicity and Vulvovaginal Candidiasis via Host Oxidative Stress Modulation and Fungal Metabolic Reprogramming

**DOI:** 10.3390/antiox14091046

**Published:** 2025-08-25

**Authors:** Yun-Dan Guo, Ming-Xuan Zhang, Quan-Yong Yu, Lu-Lu Wang, Yan-Xing Han, Tian-Le Gao, Yuan Lin, Cai Tie, Jian-Dong Jiang

**Affiliations:** 1 Institute of Materia Medica, Chinese Academy of Medical Sciences and Peking Union Medical College, Beijing 100050, China; 2 School of Pharmacy, China Pharmaceutical University, Nanjing 210009, China; 3 Institute of Medicinal Biotechnology, Chinese Academy of Medical Sciences and Peking Union Medical College, Beijing 100050, China; 4 State Key Laboratory for Fine Exploration and Intelligent Development of Coal Resources & School of Chemical and Environmental Engineering, China University of Mining and Technology (Beijing), Beijing 100083, China; tiecai@cumtb.edu.cn

**Keywords:** volatile extracts of *Hyssopus cuspidatus* Boriss, vulvovaginal candidiasis, *Candida albicans*, biofilm, riboflavin, oxidant stress

## Abstract

Background and purpose: Vulvovaginal candidiasis (VVC), caused by *Candida albicans* (*C. albicans*), is exacerbated by oxidative stress and uncontrolled inflammation. Pathogens like *C. albicans* generate reactive oxygen species (ROS) to enhance virulence, while host immune responses further amplify oxidative damage. This study investigates the antioxidant and antifungal properties of *Hyssopus cuspidatus Boriss* volatile extract (SXC), a traditional Uyghur medicinal herb, against fluconazole-resistant VVC. We hypothesize that SXC’s bioactive volatiles counteract pathogen-induced oxidative stress while inhibiting fungal growth and inflammation. Methods: GC-MS identified SXC’s major bioactive components, while broth microdilution assays determined minimum inhibitory concentrations (MICs) against bacterial/fungal pathogens, and synergistic interactions with amphotericin B (AmB) or fluconazole (FLC) were assessed via time–kill kinetics. Anti-biofilm activity was quantified using crystal violet/XTT assays, and in vitro studies evaluated SXC’s effects on *C. albicans*-induced cytotoxicity (LDH release in A431 cells) and inflammatory responses (cytokine production in LPS-stimulated RAW264.7 macrophages). A murine VVC model, employing estrogen-mediated pathogenesis and intravaginal *C. albicans* challenge, confirmed SXC’s in vivo effects. Immune modulation was assessed using ELISA and RT-qPCR targeting inflammatory and antioxidative stress mediators, while UPLC-MS was employed to profile metabolic perturbations in *C. albicans*. Results: Gas chromatography-mass spectrometry identified 10 key volatile components contributing to SXC’s activity. SXC exhibited broad-spectrum antimicrobial activity with MIC values ranging from 0.125–16 μL/mL against bacterial and fungal pathogens, including fluconazole-resistant Candida strains. Time–kill assays revealed that combinations of AmB-SXC and FLC-SXC achieved sustained synergistic bactericidal activity across all tested strains. Mechanistic studies revealed SXC’s dual antifungal actions: inhibition of *C. albicans* hyphal development and biofilm formation through downregulation of the Ras1-cAMP-Efg1 signaling pathway, and attenuation of riboflavin-mediated energy metabolism crucial for fungal proliferation. In the VVC model, SXC reduced vaginal fungal burden, alleviated clinical symptoms, and preserved vaginal epithelial integrity. Mechanistically, SXC modulated host immune responses by suppressing oxidative stress and pyroptosis through TLR4/NF-κB/NLRP3 pathway inhibition, evidenced by reduced caspase-1 activation and decreased pro-inflammatory cytokines (IL-1β, IL-6, TNF-α). Conclusions: SXC shows promise as a broad-spectrum natural antimicrobial against fungal pathogens. It inhibited *C. albicans* hyphal growth, adhesion, biofilm formation, and invasion in vitro, while reducing oxidative and preserving vaginal mucosal integrity in vivo. By disrupting fungal metabolic pathways and modulating host immune responses, SXC offers a novel approach to treating recurrent, drug-resistant VVC.

## 1. Introduction

Vulvovaginal candidiasis (VVC), a prevalent gynecological infectious disease, has emerged as a critical global public health challenge. Epidemiological studies indicate that approximately 500 million women worldwide are affected annually, with about 138 million cases progressing to recurrent VVC (RVVC), leading to significant declines in quality of life and increased mental health burdens [1,2]. The pathogenesis of VVC is closely associated with the hyphal transition of *Candida* species, with clinical manifestations including intense vulvar itching, burning pain, and in severe cases, dyspareunia, dysuria, and urinary frequency, all of which impair physiological function and social engagement [3]. Although azole antifungals (e.g., fluconazole) remain first-line therapies, the widespread emergence of drug-resistant strains (linked to drug overuse and biofilm formation) and adverse effects (e.g., hepatotoxicity, gastrointestinal disturbances) severely limit their long-term efficacy [4]. Notably, 5–10% of refractory VVC cases are attributed to azole-resistant *C. albicans* [5], underscoring the urgent need for novel, efficient, low-toxicity antifungal strategies targeting drug-resistant strains.

In recent years, bioactive compounds derived from medicinal plants have attracted significant attention due to their multifaceted mechanisms of action and reduced propensity to induce resistance compared to synthetic antimicrobials [6,7]. Among these, *Hyssopus cuspidatus* Boriss (*H. cuspidatus*), a characteristic Uyghur medicinal herb of the *Lamiaceae* family native to Central Asia and northwestern China, stands out for its traditional use in treating cold-damp respiratory disorders, attributed to its warming, expectorant, and antitussive properties [8]. While the pharmacological activities of its congener *Hyssopus officinalis* (European *hyssop*) have been extensively studied, *H. cuspidatus* exhibits distinct morphological and phytochemical profiles due to geographic divergence (European–West Asian vs. Central Asian arid zones) [9,10]. Critically, *H. cuspidatus* exhibits significantly higher pharmacological potency—particularly for anti-asthmatic effects—despite its markedly lower biomass yield (roughly 1/10 of *H. officinalis*) [11].

Previous studies demonstrate that fragrant extracts from *H. cuspidatus* exhibit superior inhibitory effects against *Staphylococcus aureus*, *Escherichia coli*, and *C. albicans* compared to its aqueous extracts [12]. However, the antifungal mechanisms of such essential oils remain poorly understood. Our research group has developed a specialized technique to optimize the extraction of volatile active components from *H. cuspidatus* (SXC), with preliminary studies revealing broad-spectrum antifungal activity, especially against *C. albicans*, including fluconazole-resistant strains. Our work specifically elucidates novel pathways—including riboflavin deprivation, Ras1-pathway suppression, and host cell protection—unexplored in earlier research.

Building on this, the present study systematically investigates the mechanisms underlying SXC oil’s efficacy against drug-resistant *C. albicans* and its therapeutic potential for VVC mice. Key research priorities include elucidating its impact on hyphal transition, biofilm formation, and modulation of host immune responses via antioxidants and inhibiting pyroptosis pathways. This study aims to provide novel insights for the development of safe and effective antifungal agents, while establishing a theoretical basis for a phytomedicine-based targeted therapeutic system for VVC.

## 2. Materials and Methods

### 2.1. Reagents and Chemicals

SXC was provided by Dingyuan Biomedical Technology (Tianjin, China). Phosphate buffered saline (PBS), cell culture media, penicillin–streptomycin, and fetal bovine serum (FBS) were obtained from Thermo Fisher Scientific (Waltham, MA, USA). Amphotericin B and fluconazole were obtained from MedChemExpress (Beijing, China).

Essential Oil Extraction Methodology: *Hyssopus cuspidatus Boriss* whole-plant material was coarsely chopped and mixed with distilled water at a ratio of 1:4–5 (*w*/*w*). The mixture was soaked at 25–30 °C for 8–10 h, after which the water was drained. The hydrated biomass underwent steam distillation using saturated steam delivered at 15–40 m/s. The resulting vapor was condensed, and the essential oil was separated from the hydrosol by decantation. The oil was dehydrated over anhydrous sodium sulfate and stored at 4 °C until use.

### 2.2. SXC Composition Analysis

A 10 µL aliquot of SXC essential oil was diluted with 990 µL of n-hexane (100-fold dilution, *v*/*v*), followed by a second 10-fold dilution (*v*/*v*) using 100 µL of the initial diluted solution mixed with 900 µL of n-hexane, yielding a total dilution factor of 1:1000 (*v*/*v*). Chromatographic separation was performed on a DB-5MS fused-silica capillary column (30 m × 0.25 mm × 0.25 µm; Agilent Technologies, Santa Clara, CA, USA) with helium (99.999% purity) as the carrier gas at a constant flow rate of 1.0 mL/min. The injection volume was 1 µL in splitless mode, and the needle wash solution was n-hexane (HEX). The oven temperature program initiated at 60 °C (held for 5 min), then increased at 4 °C/min to 100 °C (held for 2 min), followed by a 6 °C/min increase to 240 °C (held for 5 min), and finally increased at 10 °C/min to 300 °C (held for 2.67 min). Mass spectra were acquired in full-scan mode (mass range: *m*/*z* 50–500) using an electron ionization (EI) source, with the ion source temperature maintained at 250 °C, quadrupole mass analyzer at 150 °C, and transfer line temperature at 280 °C.

### 2.3. Bacterial Strains, Fungal Strains, and Growth Conditions

*C. albicans* (ATCC 64550), used in cell culture and mice infections, was purchased from the American Type Culture Collection (ATCC). *C. albicans* (ATCC 90028, ATCC 10231), *C. tropicalis* (ATCC 750), *C. krusei* (ATCC 6258), *E. coli* (ATCC 25922, ATCC 35128), *S. aureus* (ATCC 25923, ATCC 33591), *E. faecalis* (ATCC 29212), *S. epidermidis* (ATCC 12228, ATCC 51625), were also purchased from ATCC. All the bacterial strains were routinely grown in Tryptic Soy Broth (TSB) (Hope Bio-Technology, Qingdao, China) at 37 °C for 12 h at 200 rpm in the shaker (Eppendorf, Hamburg, Germany), and all the fungal strains were grown in Sabouraud Dextrose Broth (SDB) (Hope Bio-Technology, Qingdao, China) at 37 °C for 24 h at 200 rpm in the shaker.

### 2.4. Minimal Inhibitory Concentration (MIC) Determination

The antibacterial and antifungal activities of compounds were determined according to the 2021 Clinical and Laboratory Standards Institute drug sensitivity standard protocol [13]. Bacteria (1 × 10^6^ CFU/mL) were incubated with the compounds in a 96-well plate for 12 h at 37 °C. The compounds were in two-fold dilutions, with final concentrations of AmB 0.0625 μg/mL and FLC ranging from 2 to 64 μg/mL. MICs were defined as the lowest concentrations of the compounds to cause no growth of bacteria.

### 2.5. Checkerboard Assay

The synergistic effect of amphotericin B (AmB), fluconazole (FLC) and SXC was determined by performing standard checkerboard broth microdilution assays. AmB, FLC and SXC were serially diluted in eight-fold steps. Optical density 600 nm (OD_600_) was examined after incubation at 37 °C for 24 h. The FICI was calculated as shown below to analyze the synergistic effect using the concentration with the highest combination effects:

FICI = (MIC of A in the combination/MIC of A alone)+(MIC of B in the combination/MIC of B alone)

A synergistic effect is defined as FICI ≤ 0.5, while an antagonistic effect is defined as FICI ≥ 4.0. In addition, an indifferent effect is defined as 0.5 < FICI < 4.0.

### 2.6. Time–Kill Curves

Time–kill curves were examined for AmB and SXC alone or in combination for different strains at 37 °C with an initial bacterial concentration of 5 × 10^3^ CFU/mL. A 200 μL aliquot at different time points (0, 2, 4, 8, 12, 24, 48 h) was obtained and plated on Yeast Extract Peptone Dextrose Medium (YPD) agar plates after serial dilution. Fungal colonies were counted.

### 2.7. Effect of SXC on Biofilm Formation by C. albicans and Preformed Biofilms

The amount of biofilm formation was quantified with crystal violet (CV) staining [14]. Briefly, for the biofilm formation assay, *C. albicans* ATCC64550 suspension was prepared in Roswell Park Memorial Institute (RPMI) 1640 medium at a concentration of 1 × 10^6^ cells/mL with 0.25, 0.5 and 1 µL/mL concentrations of SXC, 1% Tween 80 as the control, and 200 μL added to the wells of sterile 96-well culture polystyrene plates (LABSELECT, Beijing, China). The plates were incubated statically at 37 °C, and at 12 h, 24 h, and 48 h, the medium was aspirated. The wells were washed three times with PBS (pH 6.25). Methanol (100 μL per well) was added to fix the samples for 15 min, followed by air-drying at room temperature. A 0.1% (*w*/*v*) crystal violet solution (100 μL per well) was added and incubated at room temperature for 30 min. Excess stain was removed by washing three times with PBS. Absolute ethanol (100 μL per well) was added and incubated for 1 h at 25 °C. The absorbance at 595 nm was measured using a microplate reader, with RPMI-1640 medium serving as the blank control. The biofilm formation rate was calculated using the following formula:

$$Biofilm\;formation\;rate\;\left(100\%\right)=(A595\;Drug-A595\;Blank)/(A595\;Tween\;80-A595\;Blank)$$

For the preformed biofilms, 200 μL of activated yeast cells in suspension (1 × 10^6^ cells/mL) was added to the wells of 96-well plates. After incubation at 37 °C for 24 h, the plates were washed three times with PBS. Then, 200 μL of RPMI 1640 medium containing 0.25, 0.5 and 1 µL/mL concentrations of SXC was added to the 96-well plates. After incubation at 37 °C for 24 h, the metabolic activity of the biofilm was determined with the XTT (Macklin, Shanghai, China) reduction assay [15]. The optical absorbance was measured at 450 nm on the microplate reader (BIO-RAD, iMark, Boston MA USA).

For the preformed biofilms, 1 mL of activated yeast cells in suspension (5 × 10^3^ cells/mL) was added to the wells of 12-well plates. After incubation at 37 °C for 24 h, the plates were washed three times with PBS. Then, 200 μL of RPMI 1640 medium containing 0.031, 0.062 and 0.125 µL/mL concentrations of SXC was added to the 12-well plates. After incubation at 37 °C for 24 h, a 0.1% (*w*/*v*) crystal violet solution (100 mL per well) was added and incubated at room temperature for 20 min. After discarding the staining solution, the plates were washed three times with PBS, air-dried, and then imaged under a 10× inverted microscope.

### 2.8. Cell Cultivation

The A431 cell line, obtained from Pricella, Wuhan, China, was cultured in RPMI 1640 containing 10% FBS, 100 U/mL penicillin, and 100 μg/mL streptomycin (Thermo Fisher Scientific). The Mouse Mononuclear Macrophages Cells (RAW) 264.7 cell line was provided by the Cell Resource Center of Peking Union Medical College (Beijing, China) and was cultured in DMEM containing 10% FBS, 100 U/mL penicillin, and 100 μg/mL streptomycin. All of the cells were maintained in a humidified incubator with 5% CO_2_. The cell lines were checked to be negative for mycoplasma.

### 2.9. LPS Stimulates RAW264.7 Cells

The RAW 264.7 cell suspension was inoculated into a 6-well plate at 1 × 10^5^/well, 2 mL per well for 24 h. The supernatant was discarded, and SXC was added to the culture medium containing 10% FBS DMEM to prepare 0, 1, 20 nL/mL SXC solutions. LPS was added at the same time to bring the final concentration to 750 ng/mL, with a no treatment group as a control group, for 18 h. Following the stimulation period, the cells and supernatant were collected for additional analysis to examine the effects of the different treatments.

### 2.10. Lactate Dehydrogenase (LDH) Assay

For cell infections, A431 cells (1 × 10^5^ cells/well) were seeded in 48-well plates for 12 h. *C. albicans*, grown in SDB broth to the logarithmic phase, was added to the seeded cells at a multiplicity of infection (MOI) of 10 with SXC (0, 1, 20 nL/mL) for 10 h. No infection cells were used as the control group with RPMI 1640 medium supplemented with 10% FBS. The levels of LDH released into the culture supernatants were measured using a LDH cytotoxicity assay kit (Beyotime, Shanghai, China). The absorbance signal of 490 nm was measured by a Microplate Reader (BioTek, Windsor, VT, USA).

### 2.11. Enzyme-Linked Immunosorbent Assay (ELISA)

Cytokine concentrations in the vagina were measured using mouse TNF-*α*, IL-6, IL-10, IL-1β, IL-18 and MDA ELISA kits (Bioswamp, Wuhan, China), SOD and GSH ELISA kits (NJJCBIO, Nangjing, China) according to the manufacturer’s instructions (http://www.njjcbio.com/ (accessed on 14 May 2024) and https://www.bio-swamp.com/ (accessed on 16 May 2024)). The content of SOD, GSH and MDA in the culture supernatants were measured according to the manufacturer’s instructions.

### 2.12. Gram Stanning

A431 cells were seeded at a density of 1 × 10^5^ cells/mL onto 20 mm glass coverslips placed in 12-well plates, with 1 mL per well. Cells were incubated overnight for 12 h at 37 °C, 5% CO_2_. Non-adherent cells were removed by washing the coverslips three times with DPBS. *C. albicans*, grown in SDB for 24 h, was added to the seeded cells at a multiplicity of infection (MOI) of 10 with SXC (0, 1, 20 nL/mL) for 3 h. No infection cells were used as the NC group with RPMI 1640 medium supplemented with 10% FBS. Coverslips were gently washed three times with DPBS to remove non-adherent *C. albicans*. A Gram Staining Kit (Beyotime, Shanghai, China) was used to detect *C. albicans* morphology and A431 cells according to the manufacturer’s instructions. Stained coverslips were examined under a 40× phase-contrast inverted microscope.

### 2.13. VVC Model

Female Institute of Cancer Research (ICR) 8 week old mice were obtained from Vital River Biotechnology (Beijing, China). All animals were maintained in specific pathogen free facilities under standard conditions (12 h light/12 h dark cycle, 22 ± 1 °C, 55 ± 5% relative humidity, and free access to food and water). The mice were acclimatized for 3 d before treatment.

Each mouse received a subcutaneous injection of estradiol benzoate (0.2 mg/mL) at a volume of 100 μL per mouse. Injections were administered once every 2 d for a total of three administrations. After the estradiol benzoate regimen, 60 mice were randomly selected for infection. These mice were intravaginally inoculated with 10 μL of a 30% glycerol suspension of *C. albicans* (ATCC 64550) at a concentration of 5 × 10^8^ CFU/mL. The control group received an intravaginal injection of an equivalent volume of sterile physiological saline. This inoculation procedure was performed daily for 7 consecutive days. Group Assignment and Treatment: After the 7 d inoculation period, the 60 infected mice were randomly divided into five experimental groups (n = 12 per group): model group, ciclopirox olamine group (CTZ, 150 μg/mL), fluconazole group (FLC, 150 μg/mL), SXC-L group (5 μL/mL), and SXC-H group (50 μL/mL). Each group received daily intravaginal administration of 10 μL of the respective treatment for 7 consecutive days. External vaginal signs were monitored daily throughout the treatment period. On the day 8 post-treatment initiation, vaginal lavage fluid was collected from all mice, diluted 10-fold, and analyzed using an Abbott Cell-Dyn 3700 hematology analyzer to quantify leukocyte counts. Subsequently, the mice were euthanized, and vaginal tissues were harvested for further analysis.

### 2.14. Hematoxylin and Eosin (H&E) Staining

The fixed vagina tissues were embedded in paraffin using standard techniques. Longitudinal sections of 5 μm thickness were stained with H&E stains (Servicebio, Wuhan, China) and photographed by optical microscope (Olympus CKX41, Tokyo, Japan).

### 2.15. Reverse Transcription and Quantitative Polymerase Chain Reaction (RT-qPCR)

For the mice and cells, total RNA was extracted from vagina tissues using an RNA Purification Kit (Beyotime, Shanghai, China) and then reverse transcribed with a HiFiScript cDNA Synthesis Kit for qPCR (CWBIO, Taizhou, China). Real-time PCR was performed with UltraSYBR Mixture (Low ROX) (CWBIO) on an Applied Biosystems 7500 Fast Real-Time PCR System (Applied Biosystems, Carlsbad, CA, USA). The primers used for PCR amplification are listed in Supporting Information Table S1.

For the *C. albicans*, total RNA was extracted from colon tissues using an RNA Purification Kit (Beyotime, Shanghai, China). Real-time PCR was performed with a UniPeak U+ One Step RT-qPCR SYBR Green Kit (Vazyme, Nanjing, China) on an Applied Biosystems 7500 Fast Real-Time PCR System. The primers used for PCR amplification are listed in Supporting Information Table S2. The fold changes in mRNA expressions were normalized to GraphPad Prism 9 using the ΔΔCt method.

### 2.16. Untargeted Metabolomic Analysis

For untargeted metabolomic analysis, a *C. albicans* ATCC64550 suspension was prepared in SDB at a concentration of 1 × 10^6^ cells/mL, exposed to 0, 0.125, or 0.25 µL/mL SXC essential oil, and incubated at 37 °C for 24 h. The fungal suspension was centrifuged at 6000 rpm for 5 min, and the pellet was washed three times with DPBS followed by centrifugation. To extract metabolites, 100 µL H_2_O was added to the pellet and vortexed, followed by 400 µL methanol, vortexing for 1 min, and sonication for 5 min. The mixture was centrifuged at 13,300 rpm for 10 min, and 80 µL of the supernatant was transferred to an injection vial for liquid chromatography-mass spectrometry (LC-MS) analysis. Chromatographic separation was performed on a BEH-C18-5 cm column at 30 °C with a flow rate of 0.3 mL/min and an injection volume of 3 µL. The mobile phase consisted of A (0.1% formic acid in acetonitrile) and B (0.1% formic acid in water), with the following gradient: 0–2 min (5% A, 95% B), 2–10 min (5% A to 100% A, 95% B to 0% B), 10–12 min (100% A), 12–12.1 min (100% A to 5% A), and 12.1–13.5 min (5% A). Mass spectrometry was conducted in ESI positive and negative ion modes with full-scan acquisition (50–1000 Da, 1 sec/scan, continuum data format). The scan timeline was set as 0–2 min (divert to waste), 2–12 min (direct to source), and 12–13.5 min (divert to waste).

### 2.17. Statistical Analysis

Data were processed by GraphPad Prism 10.0 software (La Jolla, CA, USA) and were presented as mean ± standard deviation (SD). One-way ANOVA was used to compare the multiple groups to evaluate the statistically significant variance. *** *p* < 0.001, ** *p* < 0.01, * *p* < 0.05 vs. model group.

## 3. Results

### 3.1. Chemical Composition Analysis of SXC via GC-MS

The chemical composition of SXC was systematically characterized using gas chromatography-mass spectrometry (GC-MS). Quantitative analysis revealed ten major constituents, which collectively accounted for 95.39% of the total mass (Table 1). The dominant components included beta-pinene (39.64%), pinocarvone (23.04%), and iso-pinocarvone (12.90%), followed by alpha-phellandrene (9.31%), limonene (2.22%), carvone (0.62%), myrtenal (3.10%), methyl myrtenyl ether (3.20%), carveol (0.86%), and methyl myrtenate (0.50%). To ensure robustness, we performed repeated analyses across multiple independent batches of SXC material. These analyses consistently revealed highly conservative chemical profiles.

Our GC-MS analysis quantified ten major constituents comprising 95.39% of the total composition, with β-pinene (39.64%), pinocarvone (23.04%), and iso-pinocamphone (12.90%) as dominant antimicrobial terpenoids. This profile aligns with literature on Xinjiang-sourced *H. cuspidatus*, confirming its characteristic high pinocarvone/β-pinene ratio—a chemotypic signature differing markedly from Mediterranean *H. officinalis*. The geographical and seasonal specificity explains SXC’s enhanced bioactivity [16]: the arid climate and flowering stage harvest optimize terpenoid synthesis, with β-pinene and pinocarvone demonstrating strong antimicrobial and anti-inflammatory effects [17].

**Figure 1:**
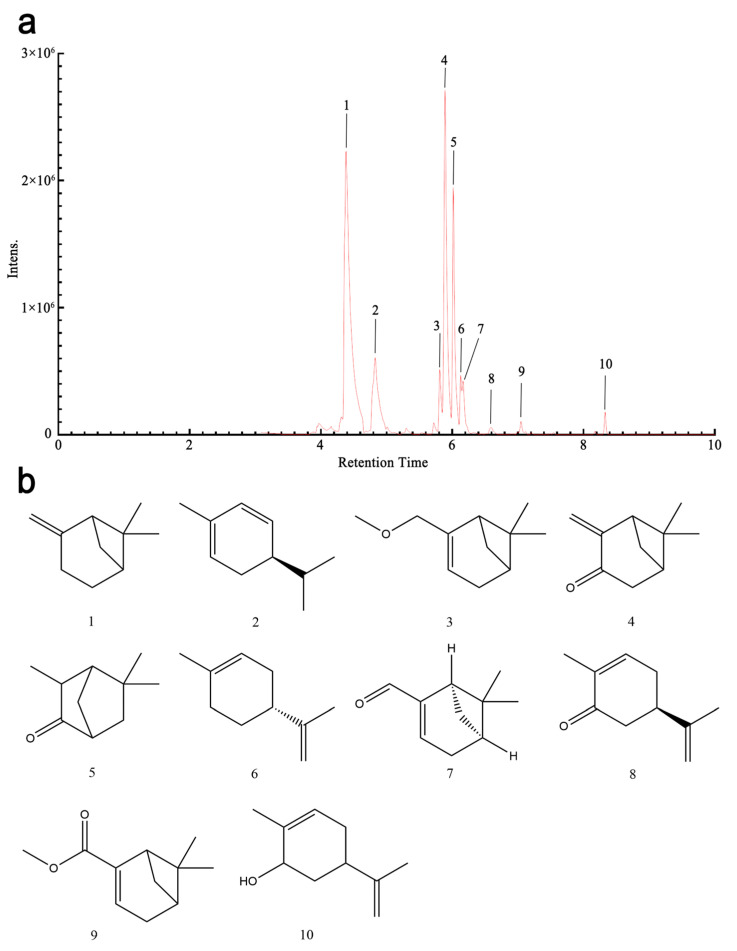
GC-MS was used to identify the main chemical components in SXC. (**a**) GC-MS chromatograms of ten compounds and a mixed standard solution of SXC. (**b**) beta-pinene, alpha-phellandrene, methyl myrtenyl ether, pinocarvone, iso-pinocarvone, limonene, myrtenal, carvone, methyl myrtenate, and carveol, pinocarvone, isopinocamphone, (+)-limonene, (+/−)-myrtenal, (−)-carvone, methyl myrtenate, and carveol. Chemical structure of the ten components.

Figure 1a presents the GC-MS chromatogram highlighting the separation and identification of these ten compounds, while Figure 1b illustrates their respective chemical structures. This comprehensive profiling provides a foundation for understanding the bioactive composition of SXC.

### 3.2. Inhibition of Bacterial and Fungal Virulence by SXC In Vitro

**Figure 2:**
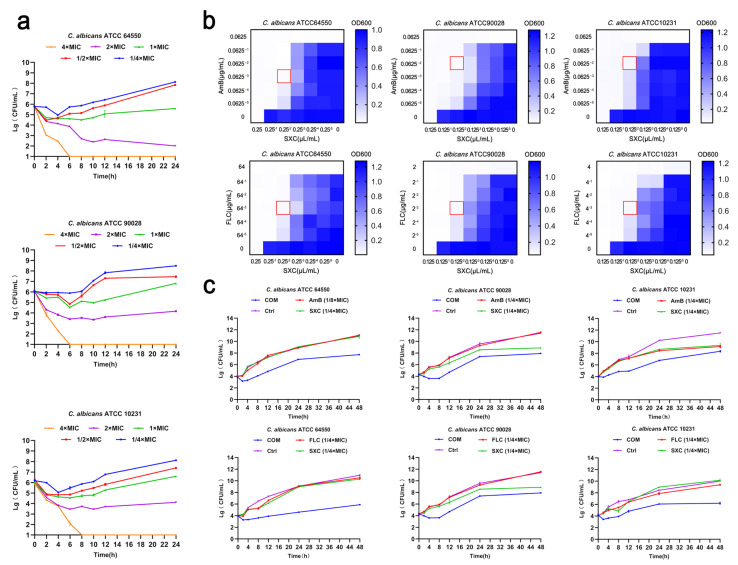
In vitro antifungal activity of AmB-SXC and FLC-SXC combinations against *C. albicans* strains. (**a**) Time–kill curves of SXC against *C. albicans* strains (n = 3). (**b**) Checkerboard analysis of AmB-SXC and FLC-SXC combinations. OD600 values were measured using a microplate reader and visualized as a color gradient (dark purple: growth; white: no growth). Red boxes indicate combinations with the highest synergistic activity. (**c**) Time–kill curves of *C. albicans* strains treated with AmB-SXC and FLC-SXC combinations at fractional inhibitory concentration index (FICI) concentrations (n = 3).

We found that SXC has a broad-spectrum anti-microbial effect. MIC of SXC against bacteria and fungi, as determined by the broth microdilution method, ranged from 0.125 to 16 µL/mL, which is rarely seen in plant purifications (Table 2). Further time–kill assays demonstrated that the bactericidal effect of SXC oil against *C. albicans* was dose-dependent, particularly for the drug-resistant strains (Figure 2a). This finding has significant clinical relevance, given the challenges in managing refractory fungal infections like *Candida*.

Checkerboard analysis was employed to assess the efficacy of antibiotic combinations compared to their individual activities, and the fractional inhibitory concentration index (FICI) was used to reflect the combined antibacterial effect. The results indicated that SXC, at concentrations of 0.031–0.062 µL/mL, could exert an antifungal-sensitizing effect on FLC and AmB, reducing the MIC of FLC and AmB to at least 1/8 of its original value (Table 3 and Table 4, Figure 2b). FICIs were calculated using combination concentrations with the highest synergistic activity, specifically the combinations of 1/8–1/4 × MIC of AmB, 1/8 × MIC of FLC, and 1/4 × MIC of SXC. Overall, all these results demonstrated that the combinations of AmB-SXC and FLC-SXC exhibited significant synergistic activity in vitro against the tested *C. albicans* strains.

Time–kill assays were conducted to investigate the synergistic effects of AmB, FLC, and SXC against all tested strains over time. The concentrations of AmB, FLC, and SXC derived from FICI analyses were utilized to construct time–kill curves. As shown in Figure 2c, monotherapy with AmB (1/8 or 1/4 × MIC), FLC (1/8 × MIC), or SXC (1/4 × MIC) induced fungal death within 0–4 h, followed by resumption of stable fungal growth after 4 h, reaching approximately 10^10^–10^11^ CFU/mL at 48 h. In contrast, the AmB–SXC and FLC–SXC combinations demonstrated pronounced growth inhibition across all three strains throughout the 0–48 h observation period. Consequently, these combinations exhibited significant synergistic activity against all tested strains, manifesting a typical bactericidal pattern with sustained suppressive effects.

### 3.3. SXC Alleviates Vaginal Lesions and Promotes Mucosal Repair in a VVC Mouse Model

**Figure 3:**
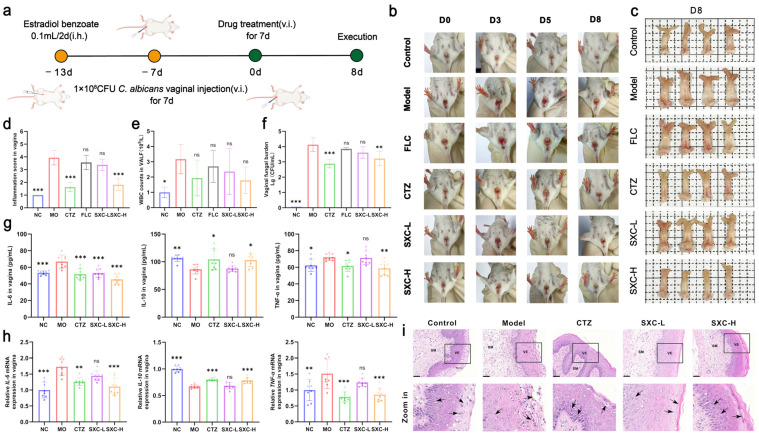
SXC attenuates vaginal damage and promotes mucosal repair in VVC mice. (**a**) Schematic diagram of the animal experimental procedure. (**b**) Representative external vaginal photographs of treated mice on day 1, day 3, day 5, and day 8 following treatment. (**c**) Vaginal tissue photographs of treated mice on day 8 post-treatment with *C. albicans* (n = 4). (**d**) Statistical analysis of external vaginal inflammation scores in treated mice on day 8 post-treatment (n = 6). (**e**) Leukocyte counts in vaginal lavage fluid of treated mice on day 8 post-treatment (n = 5). (**f**) *C. albicans* colony counts in vaginal lavage fluid of treated mice on day 8 post-treatment. (**g**) Protein expression levels of IL-10, IL-6 and TNF-αin vaginal tissues measured via ELISA (n = 8). (**h**) mRNA transcript levels of IL-10, IL-6 and TNF-α in vaginal tissues measured via RT-qPCR (n = 8). Statistical significance is indicated as follows: * *p* < 0.05, ** *p* < 0.01, *** *p* < 0.001, compared to the model group; ‘ns’ denotes not significant. (**i**) Representative histology sections stained with H&E (magnification 40×; scale bar = 20 μm). The black boxes in the upper figure indicate the regions of interest. The lower figure shows a magnified view of these boxed areas. The arrow marks the infiltration of immune cells.

We initially established a VVC mouse model and validated the protective effects of SXC, with the experimental timeline illustrated in Figure 3a. Briefly, VVC was established by priming with estradiol, followed by *Candida* inoculation 1 week before treatment, and continuous observed these animals for 8 days post drug application. We used the drug-resistant strain ATCC 64550 to mirror refractory VVC cases. This setup helps prove SXC’s efficacy in treatment-resistant scenarios. The ATCC 64550 strain is resistant to FLC, so FLC treatment had no effect (Figure 3b–f), while CTZ is effective and therefore served as a positive drug.

VVC patients typically present with substantial vaginal discharge accompanied by erythema and edema [18]. To determine whether *C. albicans* induced vaginal damage in mice, we documented vaginal morphology across all treatment groups. Photographic observations on days 1, 3, and 5 post-modeling revealed that mice in the control group maintained normal vaginal architecture without erythema, mucus secretion, ulceration, or hemorrhage. In contrast, the model group, CTZ group, FLC group, SXC-L group, and SXC-H group exhibited varying degrees of erythema, mucus secretion, ulceration, and hemorrhage (Figure 3b), which were corroborated by vaginal pathology scores and are quantified in Figure 3d. At day 8 post-treatment, vulvar erythema was substantially resolved in both the SXC-H and CTZ groups (Figure 3b). Post-euthanasia vaginal tissue dissection revealed pronounced edema in model group mice, which was alleviated to varying extents in the treatment groups (Figure 3c).

Simultaneously, ELISA and RT-qPCR assays revealed that the levels of pro-inflammatory cytokines IL-6 and TNF-α in vaginal tissues of VVC model mice were significantly elevated compared to the normal control (NC) group, while the anti-inflammatory cytokine IL-10 was markedly reduced. SXC treatment significantly reversed these alterations and alleviated vaginal inflammation in VVC mice (Figure 3g,h). We quantified leukocyte infiltration in vaginal lavage fluid. The model (MO) group demonstrated significant leukocytosis compared to NC, while the SXC-H and CTZ groups exhibited marked leukocyte reduction comparable to the MO group (Figure 3e). Concurrent analysis of fungal burden showed that the SXC-H and CTZ groups achieved significant fungal load reductions, whereas the FLC group displayed no therapeutic effect (Figure 3f).

To investigate the effects of SXC on tissue damage in VVC mice, we performed H&E staining on vaginal tissue sections. Histological examination revealed distinct morphological alterations among groups (Figure 3i). The control group exhibited intact mucosal architecture with resolution of pseudoestrus-induced squamous hyperplasia and keratinization in the vaginal epithelium (VE) [19]. In contrast, the model group demonstrated complete disruption of the mucosal layer, characterized by VE keratinization loss, epithelial desquamation, marked inflammatory cell infiltration, and concurrent mucification with submucosal (SM) edema (Figure 3i). SXC treatment demonstrated dose-dependent histopathological improvement. The SXC-H group showed partial restoration of both mucosal layer integrity and keratinized epithelial stratification, although mild residual mucification and SM edema persisted. The CTZ-treated group exhibited restored keratinization in the VE but lacked discernible mucosal layer reconstruction, with only slight SM edema remaining (Figure 3i).

### 3.4. SXC Protects Against Vaginal Mucosal Injury by Inhibiting Oxidative Stress and Pyroptosis Through the TLR4/NF-κB/NLRP3 Signaling Pathway

**Figure 4:**
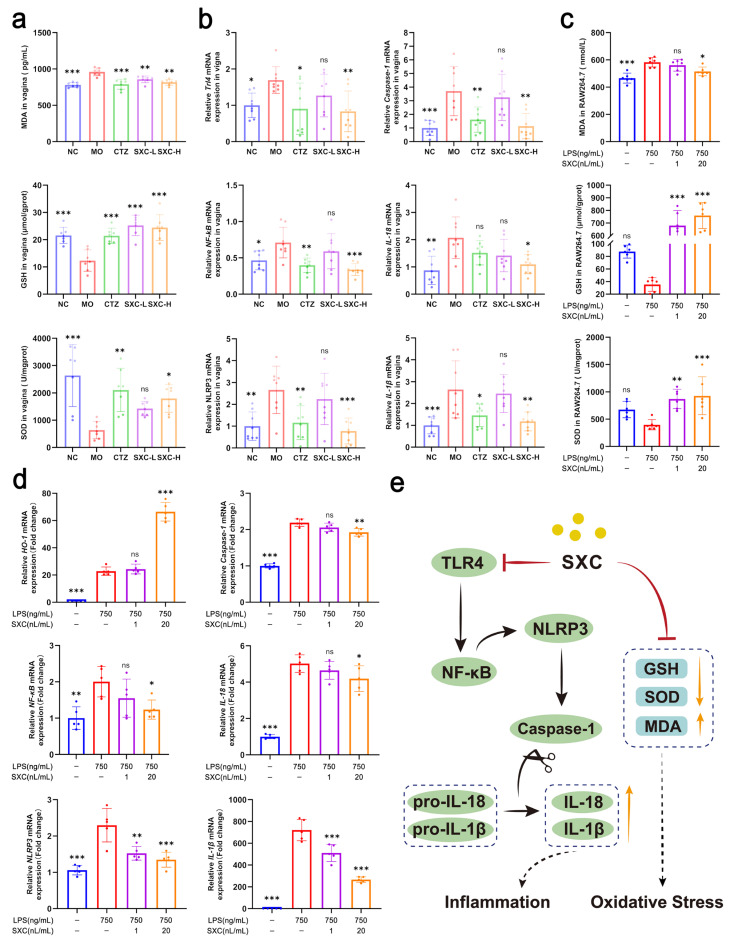
SXC treats VVC mice by exerting antioxidant effects and suppressing the NLRP3 pathway to produce anti-inflammatory actions. (**a**) Levels of GSH, SOD, and MDA in vaginal tissues (n = 7). (**b**) mRNA transcript levels of TLR4, NF-κB, NLRP3, Caspase-1, IL-18 and IL-1β in vaginal tissues measured via RT-qPCR (n = 8). (**c**) Levels of GSH, SOD, and MDA in the supernatant of LPS-stimulated RAW264.7 cells (n = 6). (**d**) mRNA transcript levels of inflammatory cytokines HO-1, NF-κB, NLRP3, Caspase-1, IL-18 and IL-1β in LPS-stimulated RAW264.7 cells (n = 6). Statistical significance is indicated as follows: * *p* < 0.05, ** *p* < 0.01, *** *p* < 0.001, compared to the model group; ‘ns’ denotes not significant. (**e**) SXC suppresses oxidative stress and pyroptosis by inhibiting the TLR4/NF-κB/NLRP3 signaling pathway.

In mucosal inflammatory diseases, excessive ROS disrupt the mucosal redox balance and induce oxidative stress, thereby promoting pathological progression [20,21]. In the VVC model, *C. albicans* invasion leads to increased cellular oxidative stress. Both in vivo and in vitro experiments demonstrated that SXC administration, compared to the model group, significantly inhibited the oxidative stress-induced elevation of MDA and reduction of GSH and SOD. This alleviated oxidative stress in vaginal and immune cells, thereby protecting cellular mitochondria and thus enhancing the antioxidant capacity of the vaginal mucosa (Figure 4a,c).

**Figure 5:**
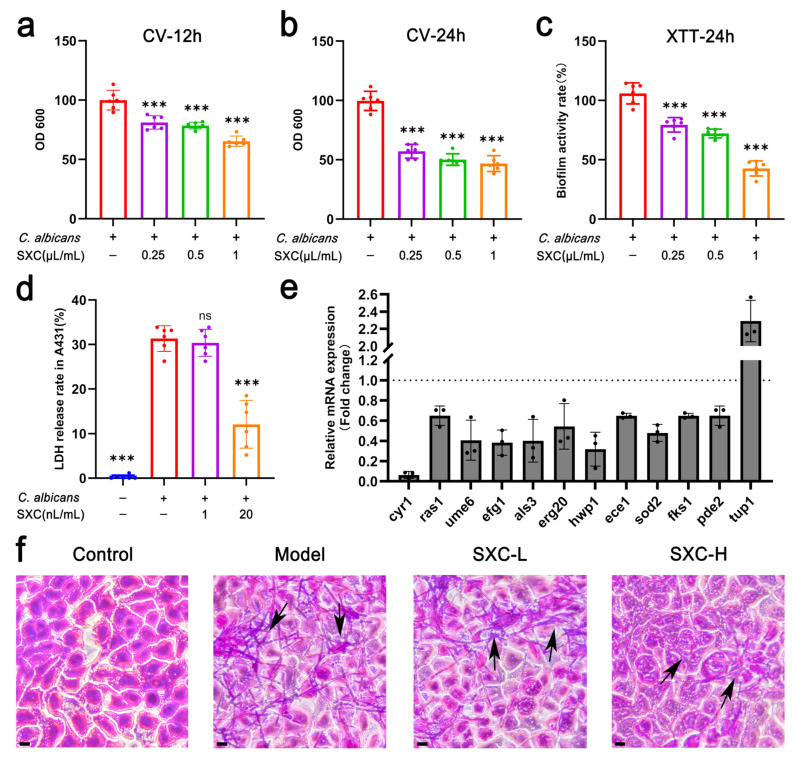
SXC inhibits *C. albicans* biofilm formation and reduces its adhesion to A431 cells. (**a**) Inhibitory effect of SXC on *C. albicans* biofilm formation at 12 h and 24 h, assessed by crystal violet assay (n = 6). (**b**) Disruptive effect of SXC on mature *C. albicans* biofilms (24 h-old), measured by crystal violet assay (n = 6). (**c**) Metabolic activity of mature *C. albicans* biofilms after SXC treatment, evaluated by XTT assay (n = 6). (**d**) LDH release rate in cell culture supernatants (n = 6). Statistical significance is indicated as follows: *** *p* < 0.001, compared to the model group; ‘ns’ denotes not significant. (**e**) Effect of SXC on biofilm-related gene expression. Expression of biofilm-associated genes was detected via quantitative reverse transcription polymerase chain reaction (RT-qPCR) (n = 3). *C. albicans* untreated with SXC served as the NC group. Gene expression was normalized to 18S rRNA levels in each sample, with NC group expression set to 1 for each gene. (**f**) Gram staining of *C. albicans* hyphae adherent to A431 cells. Scale bar = 50 μm. The arrow indicates the formation of fungal hyphae.

Upon pathogen stimulation (e.g., fungi), cytosolic pattern recognition receptors (PRRs) assemble into multiprotein complexes that induce Caspase-1 activation [22]. Activated Caspase-1 cleaves pro-IL-1β and pro-IL-18, facilitating their maturation and release [23]. Our results demonstrate that *C. albicans* infection upregulated mRNA expression of TLR4, NLRP3, Caspase-1, NF-κB, IL-1β, and IL-18 in murine vaginal tissues, which was significantly attenuated following SXC intervention (Figure 4b). We found that protein expression of IL-1β and IL-18 were downregulated in murine vaginal tissues after treatment with SXC (Supplementary Figure S1). Complementary in vitro studies confirmed that SXC modulates macrophage NLRP3 signaling by suppressing LPS-induced NF-κB and IL-1β elevation, inhibiting NLRP3 inflammasome activation, and reducing Caspase-1 and IL-18 activation (Figure 4d). Our study also revealed that SXC can stimulate the production of HO-1, but no activation of Nrf2 was observed in vitro (Figure 5b).

Collectively, these results indicate that *C. albicans* infection initiates macrophage inflammatory responses through the TLR4/NF-κB/NLRP3 pathway, amplifying tissue inflammation via cascading effects and leading to severe infection [24]. However, SXC treatment effectively interrupts this inflammatory cascade, thereby reducing tissue inflammation.

### 3.5. SXC Inhibits C. albicans Hyphae and Biofilm Formation and Attenuates its Adhesion to and Invasion of A431 Cells via the Ras1/cAMP/Efg1 Pathway

The formation of biofilms is frequently associated with the development of fungal drug resistance; consequently, inhibiting fungal biofilm formation represents a crucial strategy in antifungal drug development [25]. SXC inhibited *C. albicans* biofilm formation in a dose-dependent manner by disrupting the multi-stage process—initial adhesion (12 h), intermediate colonization (24 h), and maturation (48 h). (Figure 5a,b and Figure S4). CV staining assays revealed that SXC significantly reduced the biomass of *C. albicans* biofilms compared to the control group (Figure 5a,b). Furthermore, the XTT reduction assay demonstrated that SXC also diminished the metabolic activity of *C. albicans* biofilms (Figure 5c).

Biofilm formation encompasses multiple aspects, including adhesion capacity and hyphal development, and is regulated by numerous associated genes. To further investigate the potential antifungal mechanisms of SXC, this study evaluated its impact on the expression of biofilm-associated genes using RT-qPCR. In *C. albicans*, cAMP is synthesized by the adenylate cyclase Cyr1 [26]. Research indicates that PKA is the sole component directly downstream of cAMP and upstream of Efg1 in the cAMP-PKA signaling pathway, where it activates Efg1 through phosphorylation [27]. Among the hyphal-related genes assessed following SXC treatment, the expression of *Ras1*, *Cyr1* and *Efg1* was significantly downregulated (Figure 5e), suggesting that SXC might suppress hyphal development and biofilm formation in *C. albicans* by downregulating the Ras1-cAMP-Efg1 signaling pathway. Additionally, compared to the control group, SXC-treated samples exhibited significant downregulation of several genes involved in adhesion and hyphal formation, including *Ece1*, *Ume6*, *Pde2*, *Als3*, and *Hwp1*, while the expression of *Tup1*, a negative regulator of biofilm formation, was significantly upregulated (Figure 5e).

VVC represents an immunoinflammatory mucosal disorder caused by *C. albicans*, where adherence of the pathogen to vaginal epithelial cells initiates infection [28]. Consequently, primary therapeutic strategies for VVC focus on interfering with *C. albicans* adhesion [29]. Gram staining revealed abundant *C. albicans* hyphal formations in the model group (as compared to the NC group), while SXC treatment significantly reduced hypha-mediated adhesion to A431 epithelial cells (Figure 5f). Furthermore, LDH release assays demonstrated that SXC intervention effectively attenuated *C. albicans* invasiveness, as evidenced by significantly reduced cellular LDH release in treated groups (Figure 5d).

### 3.6. Metabolic Reprogramming in C. albicans Induced by SXC Treatment

**Figure 6:**
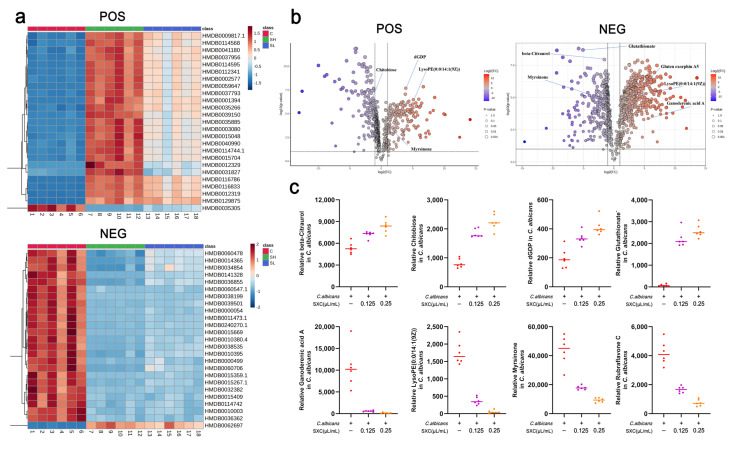
Metabolic effects of SXC on *Candida albicans*. (**a**) Metabolomics heatmap of *C. albicans* after SXC treatment (n = 6). (**b**) Metabolomics volcano plot of *C. albicans* after SXC treatment (n = 6). (**c**) Content plot of significantly different metabolites (n = 6).

Volcano plot analysis revealed profound metabolic perturbations in *C. albicans* following SXC exposure (Figure 6a). Figure 6b illustrates the differential metabolite profiling, where each data point corresponds to an individual metabolite plotted by log_2_-transformed fold change (FC, *x*-axis) and −log_10_-transformed *p*-value (*y*-axis) derived from statistical comparison between SXC-treated and untreated control groups.

Stringent statistical thresholds were applied to identify biologically relevant changes: metabolites exhibiting log_2_ FC > 1 (equivalent to FC > 2 or FC < 0.5) with *p* < 0.05 were classified as significantly dysregulated. Upregulated compounds meeting these criteria are highlighted in red, while downregulated metabolites are denoted in blue. Non-significant changes (*p* ≥ 0.05) appear as gray points clustered near the log_2_ FC = 0 baseline.

This analysis revealed that SXC induces extensive metabolic reprogramming, with multiple metabolites demonstrating statistically significant dysregulation compared to controls (Figure 6a,b). SXC treatment selectively upregulated the concentrations of glutathionate^−^, beta-Citraurol, dGDP and chitobiose, while levels of lyrsinone, rubraflavone C, LysoPE(0:0/14:1(9Z)), ganoderenic acid A and related compounds were markedly reduced (Figure 6c). Chitobiose is a key intermediate metabolite in the chitin pathway, essential for maintaining cellular morphology and environmental stress resistance in *C. albicans* [30]. Glutathionate^−^ (reduced glutathione anion, GSH^−^) functions as a core antioxidant and redox homeostasis regulator in fungal cells, to suppress the yeast-to-hyphal transition mediated by the Efg1/cAMP-PKA signaling pathway [31]. After SXC treatment, chitobiose and glutathionate^−^ levels significantly increased in *C. albicans*, suggesting that SXC may reverse the impaired hyphal formation and biofilm development (Figure 6c). LysoPE(0:0/14:1(9Z)) serves as a substrate for acyltransferase (LPLAT) and is rapidly re-acylated into intact phosphatidylethanolamine (e.g., PE (16:0/14:1)) via the Lands cycle, thereby maintaining membrane integrity [32]. Notably, SXC treatment markedly reduced levels of LysoPE(0:0/14:1(9Z)) in *C. albicans* (Figure 6c). These findings confirm that SXC exerts profound metabolic regulation through targeted modulation of specific biochemical pathways.

### 3.7. SXC Modulates C. albicans Growth Through Riboflavin Metabolic Reprogramming

**Figure 7:**
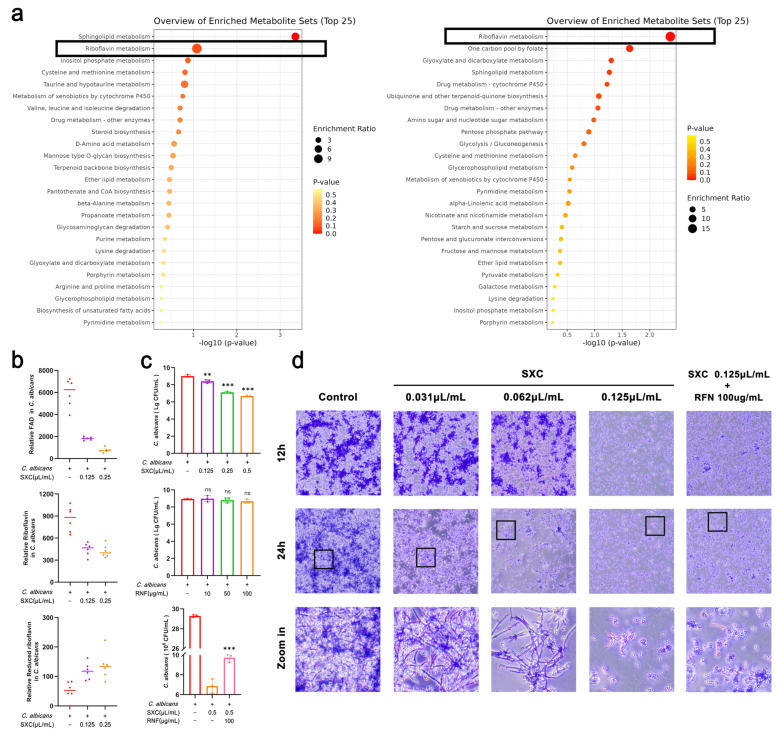
SXC modulates *C. albicans* growth and hyphal formation by affecting riboflavin metabolism. (**a**) KEGG pathway enrichment analysis of cationic and anionic metabolites in *C. albicans*. (**b**) Levels of riboflavin, reduced flavin, and flavin adenine dinucleotide (FAD) in *C. albicans* metabolites (n = 6). (**c**) *C. albicans* biomass after 24-h treatment: SXC (0.5 μL/mL), Riboflavin (100 μg/mL), SXC (0.5 μL/mL) + riboflavin (100 μg/mL) supplementation (n = 3). Statistical significance is indicated as follows: ** *p* < 0.01, *** *p* < 0.001, compared to the model group; ‘ns’ denotes not significant. (**d**) Crystal violet staining of hyphal formation: SXC (0.031, 0.062, 0.125 μL/mL) treatment groups. SXC (0.125 μL/mL) + riboflavin (100 μg/mL) rescue group. Scale bar = 100 μm.

Based on the Kyoto Encyclopedia of Genes and Genomes (KEGG) database, pathway enrichment analysis of differential metabolites detected in positive and negative ionization modes was performed. Using hypergeometric testing, the top 25 significantly enriched metabolic pathways among the differential metabolites were screened, revealing that SXC exerted a significant impact on the riboflavin metabolism pathway in *C. albicans* (Figure 7a). Further investigation demonstrated that SXC treatment significantly reduced the levels of riboflavin and FAD, while significantly increasing the level of reduced riboflavin in *C. albicans* (Figure 7b). Riboflavin is essential for FAD, and FAD is required by succinate dehydrogenase, which plays a central role in energy production within the mitochondrial respiratory chain [33]. We propose that SXC inhibits *C. albicans* growth and exerts its antifungal effect by impairing energy metabolism through the reduction of riboflavin synthesis in *C. albicans*.

Riboflavin supplementation experiments revealed that 100 µg/mL riboflavin rescued the fungicidal effect of 0.5 µL/mL SXC against *C. albicans* (Figure 7c). CV staining further showed that 100 µg/mL riboflavin rescued the growth inhibitory effect of 0.125 µL/mL SXC on *C. albicans*, although riboflavin supplementation had no significant effect on the SXC-mediated inhibition of hyphal growth (Figure 7d). Therefore, we propose that SXC inhibits *C. albicans* growth primarily by reducing riboflavin synthesis, with a lesser impact on hyphal formation.

## 4. Discussion

### 4.1. Broad-Spectrum Antimicrobial Activity of SXC Essential Oil

**Figure 8:**
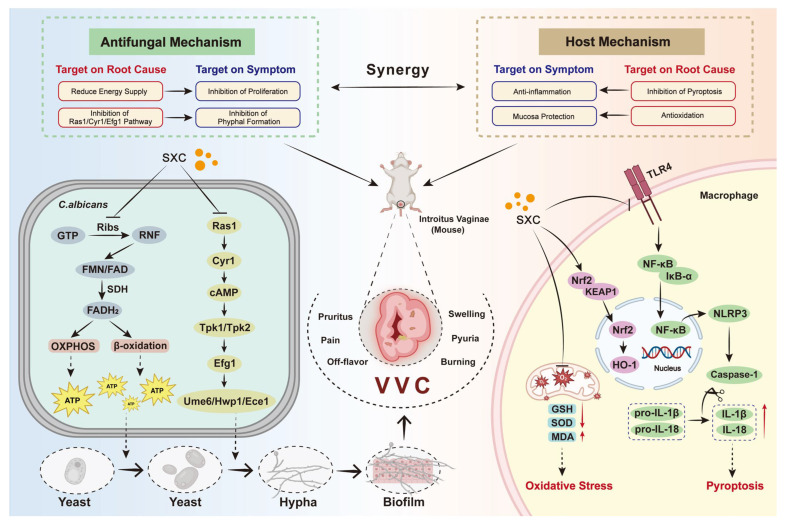
Mechanistic conclusion. SXC employs a dual therapeutic strategy against VVC by simultaneously addressing symptomatic relief and treating the root causes. For symptomatic management, SXC exhibits direct antifungal activity through inhibiting *Candida* replication, hyphal development and biofilm formation. At the same time, it mitigates host inflammation, to alleviate mucosal irritation. Beyond symptom control group, SXC targets root causes by disrupting fungal energy metabolism—reducing riboflavin availability to starve pathogens, downregulating Ras1-mediated hyphal/biofilm pathways, and counteracting oxidative stress and pyroptosis in host cells. This dual-action approach offers a comprehensive therapeutic profile that combines rapid symptom alleviation with sustained anti-relapse potential, making SXC a potential remedy for clinical refractory VVC.

This study confirms that SXC essential oil exhibits potent broad-spectrum antibacterial and antifungal activities. Notably, SCX demonstrates significant inhibitory effects against diverse bacterial strains, aligning with prior reports on its antimicrobial efficacy [12]. Its antifungal activity is particularly evidenced by effective suppression of *C. albicans* hyphal transition and biofilm formation, along with marked reduction of fungal burden in a VVC animal model. These results highlight the dual capacity of SXC oil to concurrently impede fungal proliferation and virulence-associated morphological switching. As a mechanistic conclusion, SXC employs a dual therapeutic strategy against VVC by simultaneously addressing symptomatic relief and treating the root causes (Figure 8).

### 4.2. Antifungal Mechanisms of Action

The antifungal mechanism of SXC oil combines direct fungicidal effects with profound metabolic disruption. At the cellular level, it effectively inhibits biofilm development, reduces fungal proliferation, and blocks the critical hyphal transition phase in *C. albicans*—a morphological shift essential for tissue invasion [34]. Simultaneously, SXC executes targeted metabolic sabotage by interfering with fungal energy production pathways (Figure 8).

This metabolic intervention initiates with suppression of riboflavin biosynthesis—a pathway vital for fungal survival—potentially linked to SXC-mediated inhibition of two key enzymes: riboflavin kinase and GTP cyclohydrolase [35]. Enzymatic inhibition depletes intracellular FAD and FMN, which are essential cofactors for mitochondrial respiration and fatty acid oxidation, thereby impairing lipid β-oxidation and oxidative phosphorylation, ultimately starving fungi of energy [36]. Exogenous riboflavin supplementation in *C. albicans* systems rescued colony growth but failed to restore hyphal formation defects, indicating distinct regulatory networks governing proliferation versus morphogenesis.

SXC downregulates genes in the Ras1-cAMP-Efg1 pathway, likely reducing cAMP and ATP levels, which subsequently represses hyphae-associated genes (e.g., ECE1 and HWP1). Inactivation of this pathway disrupts *C. albicans* growth, energy metabolism, yeast-to-hypha transition, and biofilm formation [37].

The metabolic perturbation induced by SXC represents a novel antifungal paradigm. By targeting fungus-specific metabolic vulnerabilities rather than conventional cellular structures, this strategy may circumvent common resistance mechanisms. Reduced FAD/FMN levels not only compromise energy metabolism but also disrupt redox homeostasis and detoxification pathways, further jeopardizing fungal survival [38].

### 4.3. Antioxidant-Derived Host Protection

VVC is characterized by superficial fungal infection, vaginal mucosal damage, and fungal bioburden [39]. In mucosae, ROS overload alters redox balance, triggering oxidative stress and signal transduction cascades that perpetuate inflammation and cellular damage [40]. This study demonstrates that SXC restores the balance between defensive and invasive factors by: suppressing oxidative stress: reducing the destructive mediator MDA while elevating protective factors SOD and GSH; modulating inflammation: inhibiting NLRP3 inflammasome activation via Toll-like receptor recognition of *C. albicans* β-glucans, thereby blocking NF-κB-mediated release of IL-1β/IL-18; and attenuating pyroptosis: preserving vaginal mucosal integrity by curtailing inflammation-associated programmed cell death [41].

In a murine vaginitis model, SXC conferred therapeutic benefits beyond direct antifungal activity, including host-protective effects. It preserved mucosal integrity while mitigating pathological immune responses: histological assessment revealed attenuated neutrophil infiltration, and vaginal lavage fluid showed diminished oxidative stress markers. This immunomodulatory profile delivers dual therapeutic advantages—resolving infection-associated tissue damage while reinforcing host defenses against fungal resistance.

### 4.4. Treatment of Both Symptoms and Root Causes

SXC demonstrates compelling antifungal potential through its dual mechanism of action: directly disrupting fungal replication while dynamically modulating host-pathogen interactions. This approach simultaneously inhibits symptoms (e.g., *Candida* replication, biofilm formation, hyphal development, and tissue inflammation) and root causes (e.g., by depriving energy production, downregulating Ras1-mediated pathways, and counteracting oxidative stress and pyroptosis in host cells). These properties align with emerging paradigms in antifungal development that prioritize synchronous targeting of microbial physiology and host-protective pathways [42]. Like many natural products with anticancer and analgesic properties [43,44], SXC shows similar broad-spectrum efficacy that targets both symptoms and underlying causes, indicating promising potential in these areas.

### 4.5. Limitations and Future Directions

Despite the significant advancements revealed in this study, several critical considerations warrant emphasis to contextualize findings and guide subsequent investigations. While SXC shows promising synergistic effects with conventional antifungals in vitro, these interactions require validation within dynamic host–pathogen interaction models—particularly in chronic infection settings. The current fungal panel, although inclusive of clinically relevant species, remains relatively restricted; expanding evaluations to multidrug-resistant and emerging fungal pathogens (e.g., *Candida auris*, azole-resistant *Aspergillus* spp.) would better establish SXC’s clinical relevance across diverse etiological contexts. Furthermore, the specific bioactive compounds within SXC and their synergistic mechanisms remain unclear and require systematic phytochemical and mechanistic profiling. As seen in studies of other natural products [45], elucidating the precise composition and functional pathways of complex natural extracts is essential to unlocking their therapeutic potential.

## 5. Conclusions

SXC emerges as a promising natural antimicrobial with broad-spectrum activity against fungal pathogens. Its multimodal mechanism of action simultaneously targets: (1) virulence attenuation through inhibition of hyphal morphogenesis, (2) metabolic disruption via riboflavin biosynthesis blockade, and (3) host immunomodulation by tempering inflammatory responses. Demonstrated efficacy across preclinical models supports its future clinical therapeutic development, particularly for topical formulations against VVC. This plant-derived agent presents a sustainable alternative to conventional antifungals, addressing the critical need for novel therapies in an era of escalating antimicrobial resistance.

## Figures and Tables

**Table 1:** Identification of chemical composition of SXC.

Serial Number	Chemical Compound	Peak Area	Aspect Ratio	CAS
1	Beta-pinene	14,484,451.87	0.3964	127-91-3
2	Alpha-phellandrene	3,403,795.41	0.0931	4221-98-1
3	Methyl myrtenyl ether	1,153,267.56	0.032	202527-57-9
4	Pinocarvone	8,419,063.42	0.2304	30460-92-5
5	Iso-pinocamphone	4,718,913.25	0.129	18358-53-7
6	(+)-Limonene	812,852.16	0.0222	5989-27-5
7	(+−)-Myrtenal	1,133,324.76	0.031	18486-69-6
8	(−)-Carvone	229,361.68	0.0062	6485-40-1
9	Methyl myrtenate	183,471.5	0.005	30649-97-9
10	Carveol	315,911.66	0.0086	99-48-9

**Table 2:** MICs of SXC.

Strains	ATCC	MIC (μL/mL)
*Staphylococcus epidermidis*	ATCC12228	0.25
*Staphylococcus epidermidis*	ATCC51625	4
*Enterococcus faecalis*	ATCC29212	8
*Staphylococcus aureus*	ATCC25923	4
*Staphylococcus aureus*	ATCC33591	8
*Escherichia coli*	ATCC25922	8
*Escherichia coli*	ATCC35128	16
*Candida albicans*	ATCC90028	0.125
*Candida albicans*	ATCC10231	0.125
*Candida albicans*	ATCC64550	0.25
*Candida tropicalis*	ATCC750	0.25
*Candida krusei*	ATCC6258	0.25

**Table 3:** MICs and FICIs of AmB with SXC against *C. albicans* strains.

MIC	Alone		Combined		FICI
	AmB (μg/mL)	SXC (μL/mL)	AmB (μg/mL)	SXC (μL/mL)	
*C. albicans* ATCC10231	0.0625	0.125	0.015	0.031	0.5
*C. albicans* ATCC90028	0.0625	0.125	0.015	0.031	0.5
*C. albicans* ATCC64550	0.0625	0.25	0.008	0.062	0.375

**Table 4:** MICs and FICIs of FLC with SXC against *C. albicans* strains.

MIC	Alone		Combined		FICI
	FLC (µg/mL)	SXC (µL/mL)	FLC (µg/mL)	SXC (µL/mL)	
*C. albicans* ATCC10231	4	0.125	0.5	0.031	0.375
*C. albicans* ATCC90028	2	0.125	0.25	0.031	0.375
*C. albicans* ATCC64550	64	0.25	8	0.062	0.375

## Data Availability

The data presented in this study are available on request from the corresponding author due to the commercial sensitivity of ongoing novel drug development at the author’s institution.

## Supplementary Materials

The following supporting information can be downloaded at: https://www.mdpi.com/article/10.3390/antiox14091046/s1, Figure S1: (a,b) Protein expression levels of IL-1β, IL-18 in vaginal tissues measured via ELISA (n = 8). (c) mRNA transcript levels of IL-6 in LPS-stimulated RAW264.7 cells (n = 6). (d) Inhibitory effect of SXC on C. albicans biofilm formation at 48 h, assessed by crystal violet assay (n = 6); Table S1: Mouse primer sequence for quantitative PCR.; Table S2: C. albicans primer sequence for quantitative PCR.

## Abbreviations

AmB	Amphotericin B
ATCC	American Type Culture Collection
CV	Crystal Violet
DMEM	Dulbecco’s Modified Eagle Medium
DPBS	Dulbecco’s Phosphate-Buffered Saline
ELISA	Enzyme-Linked Immunosorbent Assay
FAD	Flavin Adenine Dinucleotide
FBS	Fetal Bovine Serum
FICI	Fractional Inhibitory Concentration Index
FLC	Fluconazole
GC-MS	Gas Chromatography-Mass Spectrometry
GSH	Glutathione
H&E	Hematoxylin and Eosin
LDH	Lactate Dehydrogenase
LPS	Lipopolysaccharide
MDA	Malondialdehyde
MIC	Minimal Inhibitory Concentration
MO	Model Group
MOI	Multiplicity of Infection
NC	Normal Control
NLRP3	NLR Family Pyrin Domain Containing 3
PBS	Phosphate-Buffered Saline
PRRs	Pattern Recognition Receptors
ROS	Reactive Oxygen Species
RVVC	Recurrent Vulvovaginal Candidiasis
SD	Standard Deviation
SDB	Sabouraud Dextrose Broth
SOD	Superoxide Dismutase
SXC	*Hyssopus cuspidatus* Essential Oil
TLR4	Toll-Like Receptor 4
TSB	Tryptic Soy Broth
VE	Vaginal Epithelium
VVC	Vulvovaginal Candidiasis
XTT	2,3-Bis-(2-Methoxy-4-Nitro-5-Sulfophenyl)-2H-Tetrazolium-5-Carboxanilide
