# Supplementary material for: Hyssopus cuspidatus Boriss Volatile Extract (SXC): A Dual-Action Antioxidant and Antifungal Agent Targeting Candida albicans Pathogenicity and Vulvovaginal Candidiasis via Host Oxidative Stress Modulation and Fungal Metabolic Reprogramming
Source: Antioxidants (Basel). 2025 Aug 25;14(9):1046. doi: 10.3390/antiox14091046 (PMC12466673; doi:10.3390/antiox14091046)
Supplement: Supplementary file 1 [file antioxidants-14-01046-s001.zip › antioxidants-3810721-supplementary.pdf]

Supplementary Figures legends

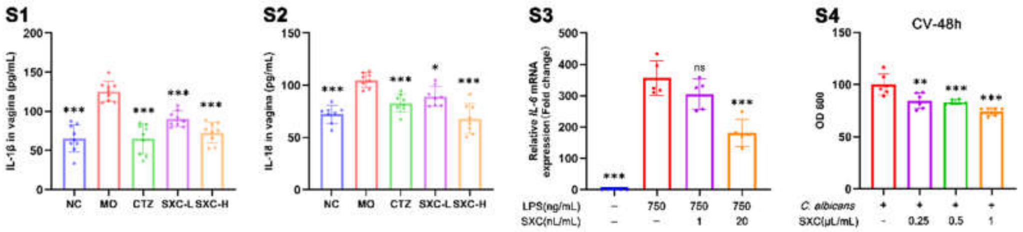

Figure S1.(a, b) Protein expression levels of IL-1β, IL-18 in vaginal tissues measured via ELISA. (n=8) (c) mRNA transcript levels of IL-6 in LPS-stimulated RAW264.7 cells. (n=6) (d)Inhibitory effect of SXC on C. albicans biofilm formation at 48 h, assessed by crystal violet assay. (n=6)

Table S1. Mouse primer sequence for quantitative PCR.

Primer	5' to 3'
GAPDH-F	GGCTGCCTTCTCTTGTGACA
GAPDH-R	TCCCGTTGATGACCAGCTTC
IL-6-F	AGCCAGAGTCCTTCAGAGAGA
IL-6-R	GGATGGTCTTGGTCCTTAGCC
TNF-α-F	CACAGAAAGCATGATCCGCG
TNF-α-R	ACTGATGAGAGGGAGGCCAT
IL-1β-F	ACTCATTGTGGCTGTGGAGA
IL-1β-R	TTGTTCATCTCGGAGCCTGT
IL-10-F	TACGTGGCTGTTACATGTAGGG
IL-10-R	GATTTGTCCGTCTGCTTTGGG
NLRP3-F	CAGACCTCCAAGACCACACTG
NLRP3-R	CATCCGCAGCAATGAACAGAG
NF-κB-F	GATCGCCACCGGATTGAAGA
NF-κB-R	CTCGGGAAGGCACAGCAATA
TLR4-F	TCTGAGCTTCAACCCCCTGA
TLR4-R	TTGTCTCAATTTCACACCTGGA
Caspase1-F	CACCCACTCGTACACGTCTT
Caspase1-R	AGGTCAACATCAGCTCCGAC
IL-18-F	TCAGCTCTTCTACCAGCAAACA
IL-18-R	CACTTCCAACTGAGAGGCTGT

Table S2. *C. albicans* primer sequence for quantitative PCR.

Primer	5' to 3'
CYR1-F	AGAAAGAAGACGATGAAACAG
CYR1-R	AGGAGAACTAGAGGATGTAGAC
EFG1-F	ACAACCTCAGCATTACAATG
EFG1-R	ATAGGTACTGCTTGTTGACC
UME6-F	TCATTCTGCTGATTTGGTCAT

UME6-R	TTGCAGCAGCACTAACACTG
HWP1-F	GCTGGCTCAAGTGGTGCTAT
HWP1-R	GGTTGCATGAGTGGAACTGA
ALS3-F	CTGGACCACCAGGAACACT
ALS3-R	ACCTGGAGGAGCAGTGAAAG
ECE1-F	GCTGGTATCATTGCTGATAT
ECE1-R	TTCGATGGATTGTTGAACAC
RAS1-F	GTGGTGGTGTTGGTAAATC
RAS1-R	TTCTTGTCCAGCAGTATCT
SOD2-F	AAACTTGGCTCCTGTCTC
SOD2-R	GTATCACCATTGGCTTTG
ERG1-F	CTCCTTCTGCTGCTAACG
ERG1-F	CCAACTGTCATACCACCC
β-actin-R	ACCGAAGCTCCAATGAATCC
β-actin-R	CCGGTGGTTCTACCAGAAGAG
PDE-F	TGCTGTGGGACATTGGAG
PDE-R	GGCGGAAATTATGGAACG
ERG20-F	TTACCCGTGGCATTAGCAATGTA
ERG20-R	TCCCAAGGGAATCAAAATGTCTC
FKS1-F	CGTGAAATTGATCATGCCTGTAC
FKS1-R	AACCCTTCTGGGCTCCAAA
TUP1-F	CTTGGAGTTGGCCCATAGAA
TUP1-R	TGGTGCCACAATCTGTTGTT
